# Effects of Hypoxia-Inducible Factor Prolyl Hydroxylase Inhibitors on Relationship Between B-Type Natriuretic Peptide and Hemoglobin Levels in Patients With Cardiorenal Anemia Syndrome

**DOI:** 10.1155/cdr/3143864

**Published:** 2025-11-06

**Authors:** Tomoaki Yoshitake, Toru Hashimoto, Shouji Matsushima, Kei Ikuta, Shoei Yamamoto, Tomoyasu Suenaga, Shunsuke Nakashima, Takashi Kai, Kayo Misumi, Keisuke Shinohara, Takeo Fujino, Shunsuke Katsuki, Kazuya Hosokawa, Shintaro Kinugawa, Kohtaro Abe

**Affiliations:** ^1^Department of Cardiovascular Medicine, Faculty of Medical Sciences, Kyushu University, Fukuoka, Japan; ^2^Department of Advanced Cardiopulmonary Failure, Faculty of Medical Sciences, Kyushu University, Fukuoka, Japan

**Keywords:** anemia, cardiorenal syndrome, chronic kidney disease, heart failure, hypoxia-inducible factor prolyl hydroxylase inhibitors, iron deficiency

## Abstract

**Backgrounds:**

Anemia is associated with poor prognosis in heart failure patients. Erythropoiesis-stimulating agents have been used for the treatment of renal anemia, but they increase the risk of thrombotic events. Hypoxia-inducible factor prolyl hydroxylase inhibitors (HIF-PHIs) have emerged as a new treatment for renal anemia. HIF-PHIs are expected to have pleiotropic effects beyond correcting anemia. Evidence regarding the effects of HIF-PHIs in cardiorenal anemia syndrome has not been established.

**Methods and Results:**

The present study is a single-center, retrospective analysis of patients with heart failure and renal anemia who were treated with HIF-PHIs or oral iron supplementation at Kyushu University Hospital (*n* = 39 and *n* = 26). Both treatments significantly raised hemoglobin levels (HIF-PHIs 9.1 [8.6–9.6] vs. 10.3 [9.5–11.8], *p* < 0.001; oral iron 10.1 [9.3–11.0] vs. 11.8 [10.6–13.2], *p* < 0.001). Log-transformed BNP level (logBNP) decreased in the HIF-PHI-treated patients but not in those treated with oral iron despite the similar improvement in hemoglobin levels (HIF-PHIs 2.74 [2.28–2.99] vs. 2.47 [2.16–2.66], *p* = 0.005; oral iron 2.38 [1.89–2.66] vs. 2.37 [1.90–2.50], *p* = 0.711). The levels of iron metabolism–related parameters after treatment were comparable between the two groups. ΔlogBNP/ΔHb slope was significantly steeper in the HIF-PHI group than the oral iron supplementation group (−0.100 vs. −0.047, *p* = 0.039), meaning that HIF-PHIs further reduced BNP levels than anticipated by the increase of hemoglobin with conventional treatment.

**Conclusion:**

HIF-PHIs reduce BNP levels more than anticipated by the elevation of hemoglobin levels compared to oral iron supplementation. HIF-PHIs may have therapeutic benefits against heart failure beyond the correction of anemia in the heart failure patients.

## 1. Introduction

Anemia is common and an independent predictor of poor prognosis in patients with heart failure [1, 2]. The causes of heart failure–associated anemia include nutritional deficiencies, chronic inflammation, chronic kidney disease (CKD), gastrointestinal bleeding, hematologic diseases, and hemodilution [3]. Iron deficiency is present in 50% of patients with heart failure with reduced ejection fraction (HFrEF). Although oral iron supplementation did not improve exercise capacity, intravenous ferric carboxymaltose therapy improves exercise capacity in patients with heart failure and iron deficiency [4–9]. Erythropoiesis-stimulating agents (ESAs) have been used for the treatment of renal anemia, and they improve exercise tolerance in patients with heart failure [10]. However, darbepoetin-alpha did not reduce death or heart failure hospitalization but rather increased ischemic stroke and thrombotic events in HFrEF patients, raising safety concerns about the use of ESAs in heart failure patients [11].

Hypoxia-inducible factor prolyl hydroxylase inhibitors (HIF-PHIs) have emerged as a new treatment for renal anemia. HIF is a master transcriptional regulator of the cellular responses to hypoxia [12]. Roxadustat, vadadustat, daprodustat, molidustat, enarodustat, and desidustat have shown noninferiority to ESAs in increasing hemoglobin levels in patients with dialysis-dependent (DD) and non–dialysis-dependent (NDD) CKD [13–23]. HIF-PHIs also improve iron metabolism [24]. HIF-PHIs have beneficial therapeutic potential against heart failure by correcting anemia and improving iron metabolism, but evidence regarding the effects of HIF-PHIs in cardiorenal anemia syndrome has not been established. We sought to evaluate the therapeutic effects of HIF-PHIs in patients with heart failure complicated by renal anemia in the present study.

## 2. Methods

This study is a single-center, retrospective analysis of the consecutive patients diagnosed with heart failure and renal anemia who were newly administered HIF-PHIs or oral iron supplementation at the Department of Cardiovascular Medicine, Kyushu University Hospital, from 2020 through 2024. The selection of the treatment drugs for anemia depended on each physician's decision. Clinical data were obtained from the patients' medical records. Renal anemia was defined as hemoglobin < 13 g/dL for male and < 12 g/dL for female patients with estimated glomerular filtration rate (eGFR) < 60 mL/min/1.73 m [2]. We compared the drug effects between the patients treated with HIF-PHIs and those treated with oral iron supplementation. This study was conducted according to the principles of the Declaration of Helsinki. The study protocol was approved by the Institutional Review Board (#21113-01). Patients were offered the opportunity to opt out of the study. The data were presented as median with interquartile range. Continuous variables were compared with a paired *t*-test or Wilcoxon test for paired samples and an unpaired *t*-test or Mann–Whitney test for unpaired samples. Categorical values were compared with Fisher's exact test. Univariate and multiple linear regression analyses were performed for describing the relationship. The difference of linear regression slopes was tested by the comparison of least square means with Dunnett's method. Significance was defined as *p* < 0.05. Statistical analysis was performed using JMP Pro Version 17.0.0 (JMP Statistical Discovery LLC, Cary, North Carolina, United States).

## 3. Results

We identified 65 patients with chronic heart failure accompanied by renal anemia who were newly administered HIF-PHIs or oral iron supplementation (HIF-PHIs *n* = 39, oral iron *n* = 26). The baseline characteristics are shown in Table 1. The hemoglobin level and eGFR were lower in the HIF-PHI group than oral iron group. The levels of serum iron, ferritin, and transferrin saturation (TSAT) were lower in the oral iron–supplemented patients than in the HIF-PHI-treated patients. The etiologies and severity of heart failure were not significantly different between the two groups. Left ventricular ejection fraction (LVEF), left ventricular dimensions, and medications for heart failure were comparable between the two groups.

**Figure 1:**
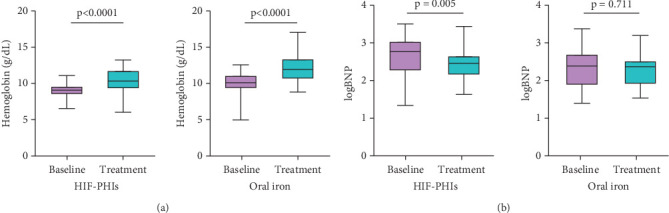
Treatment effects of hypoxia-inducible factor prolyl hydroxylase inhibitors and oral iron supplementation on hemoglobin and logBNP levels in patients with chronic heart failure associated with renal anemia. The levels of (a) hemoglobin and (b) logBNP at baseline and after the treatment with each drug are shown. Values are presented as median with interquartile range (first and third quartiles). *p* values for paired *t*-test.

The median follow-up duration was 267 (170–460) days in the HIF-PHI group and 253 (105–552) days in the oral iron supplementation group. There was no statistically significant difference in the follow-up periods between groups (*p* = 0.896). Both HIF-PHIs and oral iron supplementation significantly raised hemoglobin levels (Figure 1a). The change in hemoglobin levels (ΔHb) was not significantly different between groups (*p* = 0.153). Interestingly, log-transformed BNP level (logBNP) significantly decreased in the HIF-PHI-treated patients but not in the oral iron–treated patients despite the similar improvement in hemoglobin levels (Figure 1b). The serum levels of iron metabolism–related parameters were comparable between the two groups (Table 2). The difference of drugs in the HIF-PHI class was not correlated with the change in hemoglobin (*p* = 0.286) and logBNP (*p* = 0.217). The use of medications for heart failure and thrombosis (angiotensin-converting enzyme inhibitor [ACEI], angiotensin receptor blocker [ARB], angiotensin receptor–neprilysin inhibitor [ARNI], *β*-blockers, mineralocorticoid receptor antagonist [MRA], SGLT2 inhibitors, diuretics, antiplatelets, and oral anticoagulants) was not significantly correlated with the change in logBNP (ΔlogBNP) and ΔHb. Systolic and diastolic blood pressure (SBP and DBP) did not significantly elevate after the treatment (HIFPHIs: *p* = 0.339 for SBP, *p* = 0.792 for DBP; oral iron: *p* = 0.716 for SBP, *p* = 0.913 for DBP). We did not observe any adverse events such as thrombotic events, stroke, and malignancies during the follow-up period, regardless of the HIF-PHI types. No statistically significant difference was observed in the prescription rates of drugs for heart failure (ACEI/ARB/ARNI, *β*-blockers, MRAs, SGLT2 inhibitors, and diuretics), oral anticoagulants, and antiplatelets between baseline and follow-up, either in the HIF-PHI group or the oral iron supplementation group. This suggests that the changes in other medications during the observation period did not affect the results (Table 3).

**Figure 2:**
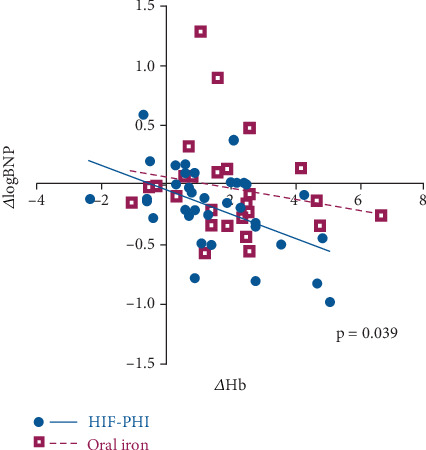
The relationship between the changes in levels of logBNP and hemoglobin. The ΔlogBNP/ΔHb slopes by linear regression were compared between the patients treated with HIF-PHIs and those treated with oral iron supplementation (*p* = 0.039 for Dunnett's test). HIF-PHIs, *n* = 39. Oral iron supplementation, *n* = 26.

As shown in Table 4, the univariate analysis showed that the baseline levels of eGFR and hemoglobin level were significantly or nearly significantly correlated with ΔlogBNP. The 95% confidence interval (CI) and *p* values were as follows: age (−0.004 to −0.011, *p* = 0.378), sex (*p* = 0.611), eGFR (0.000–0.014, *p* = 0.056), hemoglobin (0.056–0.187, *p* = 0.0006), serum iron (−0.002 to 0.002, *p* = 0.699), ferritin (−0.0001 to 0.0003, *p* = 0.428), TSAT (−0.007 to 0.004, *p* = 0.555), and LVEF (−0.003 to 0.007, *p* = 0.402). The multivariate analysis incorporating age, sex, eGFR, and hemoglobin level at baseline revealed that the baseline hemoglobin level was the only independent predictor of ΔlogBNP (0.015–0.270, *p* = 0.036). Since the relationship between ΔlogBNP and ΔHb (ΔlogBNP/ΔHb) was not correlated with the baseline hemoglobin level (*p* = 0.107), we evaluated ΔlogBNP/ΔHb thereafter. ΔlogBNP/ΔHb slope was significantly steeper in the HIF-PHI group than the oral iron supplementation group (−0.100 vs. −0.047, *p* = 0.039, Figure 2). This finding means that HIF-PHIs further reduced BNP levels than anticipated by the increase of hemoglobin with conventional treatment, suggesting the additional benefits of HIF-PHIs beyond correcting anemia in patients with chronic heart failure. The types of HIF-PHIs did not significantly affect ΔlogBNP/ΔHb slopes (*p* = 0.549).

## 4. Discussion

In the present study, we demonstrated that both HIF-PHIs and oral iron supplementation improved anemia to a similar extent in patients with heart failure accompanied by renal anemia. The levels of iron metabolism–related parameters were comparable between the two groups after the treatment. Interestingly, the significant decline of logBNP was observed only in the HIF-PHI-treated patients. ΔlogBNP/ΔHb slope was significantly steeper in the HIF-PHI-treated patients than those treated with oral iron, meaning that HIF-PHIs further reduced BNP levels than anticipated by the rise of hemoglobin levels and improvement of iron metabolism. We believe that this is the first report demonstrating the additional benefits of HIF-PHIs beyond correcting anemia in patients with heart failure. This effect may be attributable to pleiotropic effects of HIF-PHIs such as HIF-mediated control of cell survival/growth, energy metabolism, epigenetic regulation, oxidative stress responses, inflammation, and endothelial functions; but further investigation is required to elucidate the mechanism [25, 26].

The levels of natriuretic peptides are determined by various factors such as hemodynamic overload, heart rate, atrial arrhythmia, age, body mass index, renal function, and anemia [27–29]. The plasma level of natriuretic peptides decreases as the level of hemoglobin increases [29]. Although we did not observe a significant decline in BNP levels in the oral iron–treated patients despite the rise in hemoglobin levels, it might have required a greater increase in hemoglobin levels than observed to see the decline of BNP at least in the present cohort. The observation that treatment of anemia with HIF-PHIs significantly reduced the levels of BNP even in this patient population suggests an advantage of HIF-PHIs over treatment with oral iron supplementation, although its mechanism remains to be elucidated. There have been few studies reporting on the effects of HIF-PHIs in patients with heart failure. Some studies did not observe a reduction of BNP or NT-proBNP despite an increase in hemoglobin levels by the treatment with HIF-PHIs in patients with heart failure and renal anemia [30–32]. Another study demonstrated that vadadustat significantly decreased serum NT-proBNP levels and improved NYHA functional class in patients with heart failure and renal anemia [33]. This discrepancy may be attributable to small sample sizes, differences in the background characteristics of patients, and/or inadequate improvement of anemia.

ESAs have been used for the treatment of renal anemia for decades. However, there have been concerns about thrombotic risk with the use of ESAs in patients with HFrEF [11]. HIF-PHIs have recently emerged as a new class of agents for the treatment of renal anemia. When activated, HIFs regulate hundreds of genes involved in erythropoiesis, angiogenesis, lipid and glucose metabolism, glycolysis, mitochondrial function, cell growth and survival, vasodilation, and cell migration [34]. HIF-PHIs also improve iron metabolism and availability [24]. HIF-PHIs are expected to be effective alternatives to ESA and also to have erythropoiesis-independent pleiotropic effects such as anti-inflammatory and lipid-lowering effects [34, 35]. However, the cardiovascular safety concerns are still controversial. A meta-analysis including 30,994 NDD-CKD and DD-CKD patients showed no difference between HIF-PHIs and ESAs for major adverse cardiovascular events [36]. In the post hoc analysis of ASCEND-ND and ASCEND-D trials, daprodustat increased total heart failure hospitalizations in NDD-CKD but not in DD-CKD patients, although it did not significantly raise the risk of the composite outcome of first heart failure hospitalization or death [37]. Some HIF-PHIs raise the risk of arteriovenous access thrombosis and venous thromboembolism [36]. Meta-analyses have shown no significant differences in the development of hypertension between HIF-PHIs and ESAs [36]. Based on our observation and previous reports, we believe that HIF-PHIs have protective effects against heart failure, as long as they are used in well-managed heart failure patients [33]. Further studies are needed to identify the suitable population who will benefit from HIF-PHIs among patients with heart failure.

This study has several limitations. First, this was a retrospective and observational study with a small sample size from a single center. Therefore, the results may not be applicable to all heart failure population. In addition, the start of HIP-PHIs or oral iron supplementation depended on each physician's decision, so there might be a selection bias. Future prospective, controlled, and randomized studies are necessary. Second, the control arm is not the nontreatment population but the patients treated with iron supplementation. Our aim is to demonstrate the therapeutic benefits of HIF-PHIs beyond improvement of anemia, so the control arm should have been treated with a conventional intervention to correct anemia. We did not adopt ESA-treated patients as a control group, given the advantage of HIF-PHIs as oral medications. However, a future head-to-head investigation is needed to demonstrate the advantage of HIF-PHIs over ESAs definitely. Lastly, the baseline characteristics were significantly different between the two groups. But the propensity score matching was difficult due to the small sample size. Instead, we evaluated ΔlogBNP/ΔHb, which was independent of the baseline hemoglobin level, and demonstrated a significant drug effect of HIF-PHIs on the ΔlogBNP/ΔHb slope. We believe that this finding offers the basis of pursuing the clinical research of HIF-PHIs in patients with heart failure. Future investigation including more patients from multicenters with an appropriate control arm is required.

In conclusion, HIF-PHIs reduce BNP levels more than anticipated by the correction of hemoglobin levels compared to oral iron supplementation. HIF-PHIs may have therapeutic benefits against heart failure beyond the correction of anemia in the heart failure patients.

## Figures and Tables

**Table 1:** Baseline characteristics of patients with cardiorenal anemia syndrome.

	**HIF-PHIs ( ** **n** = 39** )**		**Oral iron ( ** **n** = 26** )**		**p** ** value**
	**Median or number**	**[IQR] or (%)**	**Median or number**	**[IQR] or (%)**	
Age (years)	67	[56, 77]	68	[54, 74]	0.679
Female sex	19	(50.0)	11	(42.3)	0.615
Follow-up duration (days)	267	[170, 460]	253	[105, 552]	0.896
Body mass index (kg/m^2^)	21	[19, 23]	22	[20, 24]	0.332
Etiologies of heart failure					0.1123
Dilated cardiomyopathy	8	(20.5)	4	(15.4)	
Valvular heart disease	8	(20.5)	3	(11.5)	
Hypertrophic cardiomyopathy	6	(15.4)	1	(3.8)	
Ischemic heart disease	5	(12.8)	7	(26.9)	
ATTR amyloidosis	4	(10.3)	1	(3.8)	
Hypertensive heart disease	2	(5.1)	0	(0)	
Cardiac sarcoidosis	0	(0)	3	(11.5)	
Others	6	(15.4)	7	(26.9)	
Comorbidities					
Hypertension	13	(33.3)	7	(26.9)	0.784
Diabetes	8	(20.5)	11	(42.3)	0.094
Atrial fibrillation	23	(59.0)	13	(50.0)	0.611
Hemodialysis	1	(2.6)	0	(0)	0.385
History of stroke	9	(23.1)	5	(19.2)	0.768
History of VTE	3	(7.7)	1	(3.8)	0.644
History of malignancy	4	(10.3)	2	(7.7)	> 0.99
NYHA functional class					0.621
I	6	(15.4)	3	(3.8)	
II	12	(30.8)	9	(46.2)	
III	12	(30.8)	11	(42.3)	
IV	9	(23.1)	3	(3.8)	
SBP (mmHg)	96	[83, 109.5]	103	[88.5, 119.8]	0.157
DBP (mmHg)	60	[53, 69.5]	61	[51.3, 69.8]	0.854
Laboratory tests					
Creatinine (mg/dL)	1.62	[1.41, 2.03]	1.2	[1.06, 1.55]	0.0001
eGFR (mL/min/1.73 m^2^)	28.6	[23, 35.9]	39.2	[32.7, 48.3]	0.002
Serum iron (*μ*g/dL)	65	[45, 83]	39	[27, 52]	0.009
Ferritin (ng/mL)	230.8	[88.0, 400.7]	23.9	[16.6, 86.4]	< 0.0001
UIBC (*μ*g/dL)	209	[167, 248]	303	[204, 346]	0.004
TSAT (%)	23.3	[19.2, 31.2]	10.8	[8.9, 21.1]	0.001
Hemoglobin (g/dL)	9.0	[8.7, 9.5]	10.1	[9.3, 10.9]	0.006
BNP (pg/mL)	447.5	[184.6, 830.7]	242.0	[81.0, 425.7]	0.075
Echocardiography					
LVIDD (mm)	47.0	[42.0, 57.5]	47.5	[42.0, 54.6]	0.961
LVIDS (mm)	36.5	[28.0, 45.8]	36.5	[27.0, 44.8]	0.580
LVEF (%)	50.9	[33.8, 63.1]	49.1	[27.0, 64.7]	0.865
Medications					
*β*-Blockers	24	(61.5)	19	(73.1)	0.426
ACEI/ARB	15	(38.5)	15	(57.7)	0.139
ARNI	3	(7.7)	2	(7.7)	> 0.99
MRAs	16	(41.0)	11	(42.3)	> 0.99
SGLT2 inhibitors	14	(35.9)	12	(46.2)	0.448
Diuretics	31	(79.5)	15	(57.7)	0.094
Anticoagulants	25	(64.1)	21	(80.8)	0.174
Antiplatelets	12	(30.8)	9	(34.6)	0.791
HIF-PHIs					
Daprodustat	15	(38.5)			
Roxadustat	14	(35.9)			
Vadadustat	8	(20.5)			
Molidustat	2	(5.1)			

Abbreviations: ACEI, angiotensin-converting enzyme inhibitor; ARB, angiotensin receptor blocker; ARNI, angiotensin receptor–neprilysin inhibitor; BNP, B-type natriuretic peptide; BUN, blood urea nitrogen; DBP, diastolic blood pressure; eGFR, estimated glomerular filtration rate; HIF-PHI, hypoxia-inducible factor prolyl hydroxylase inhibitor; LVEF, left ventricular ejection fraction; LVIDD, left ventricular internal dimension at diastole; LVIDS, left ventricular internal dimension at systole; MRA, mineralocorticoid receptor antagonist; NYHA, New York Heart Association; SBP, systolic blood pressure; SGLT2, sodium–glucose transporter-2; TSAT, transferrin saturation; UIBC, unsaturated iron-binding capacity; VTE, venous thromboembolism.

**Table 2:** Posttreatment iron metabolism parameters.

	**HIF-PHIs ( ** **n** = 39** )**		**Oral iron ( ** **n** = 26** )**		**p** ** value**
	**Median**	**[IQR]**	**Median**	**[IQR]**	
Hemoglobin (g/dL)	10.2	[9.4, 11.4]	11.8	[10.75, 13.1]	0.0009
Serum iron (*μ*g/dL)	75	[57, 113.3]	73.5	[52.8, 115.3]	0.616
Ferritin (ng/mL)	126.6	[92, 330.4]	80.2	[45.2, 237.3]	0.143
UBIC (*μ*g/dL)	222	[159, 287]	206	[164.5, 239]	0.24
TSAT (%)	25.9	[17.7, 43.9]	23.6	[20.0, 40.0]	0.982

Abbreviations: TSAT, transferrin saturation; UIBC, unsaturated iron-binding capacity.

**Table 3:** Medications at baseline and at follow-up.

	**HIF-PHI ( ** **n** = 39** )**		**p** ** value**	**Oral iron ( ** **n** = 26** )**		**p** ** value**
	**Baseline**	**Follow-up**		**Baseline**	**Follow-up**	
	**N** ** (%)**	**N** ** (%)**		**N** ** (%)**	**N** ** (%)**	
ACEI/ARB/ARNI	15 (38.5)	16 (41.0)	> 0.999	19 (73.1)	18 (69.2)	> 0.999
*β*-Blockers	21 (53.9)	23 (59.0)	0.82	21 (80.8)	19 (73.1)	0.743
MRAs	14 (45.2)	17 (43.6)	0.644	14 (53.9)	15 (57.7)	> 0.999
SGLT2 inhibitors	11 (28.2)	17 (43.6)	0.238	13 (50.0)	14 (53.9)	> 0.999
Diuretics	27 (69.2)	27 (69.2)	> 0.999	17 (65.4)	16 (61.5)	> 0.999
Anticoagulants	22 (56.4)	21 (53.9)	> 0.999	22 (84.6)	21 (80.8)	> 0.999
Antiplatelets	11 (28.2)	9 (23.1)	0.796	9 (34.6)	6 (23.1)	0.541

Abbreviations: ACEI, angiotensin-converting enzyme inhibitor; ARB, angiotensin receptor blocker; ARNI, angiotensin receptor–neprilysin inhibitor; HIF-PHI, hypoxia-inducible factor prolyl hydroxylase inhibitor; MRA, mineralocorticoid receptor antagonist; SGLT2, sodium–glucose transporter-2.

**Table 4:** Univariate and multivariate analysis of the change in logBNP (ΔlogBNP).

**Variables**	**Regression coefficient**	**95% CI**	**R** ^2^	**p**	**Regression coefficient**	**95% CI**	**p**
Age (years)	0.0033	−0.004, 0.011	0.0126	0.378	0.0004	−0.009, 0.010	0.939
Sex	−0.0291	−0.140, 0.082	0.0072	0.611	-0.0328	−0.145, 0.080	0.571
eGFR, (mL/min/1.73 m^2^)	0.007	0.000, 0.014	0.0576	0.056	0.0038	−0.006, 0.014	0.473
Serum iron (*μ*g/dL)	0.0004	−0.002, 0.002	0.0026	0.699			
Ferritin (ng/mL)	0.0001	−0.0001, 0.0003	0.0112	0.428			
TSAT (%)	−0.0016	−0.007, 0.004	0.006	0.555			
Hemoglobin (g/dL)	0.1213	0.056, 0.187	0.1763	0.0006	0.1426	0.015, 0.270	0.036
LVEF (%)	0.002	−0.003, 0.007	0.0116	0.402			

Abbreviations: BNP, B-type natriuretic peptide; eGFR, estimated glomerular filtration rate; LVEF, left ventricular ejection fraction; TSAT, transferrin saturation.

## Data Availability

The deidentified participant data will not be shared.
